# Chronic inflammatory demyelinating polyneurophaty: assessment of the cognitive function and quality of life

**DOI:** 10.1055/s-0042-1758455

**Published:** 2022-12-29

**Authors:** Larissa Kozow, Camila Pupe, Osvaldo J. M. Nascimento

**Affiliations:** 1 Universidade Federal Fluminense, Hospital Universitário Antônio Pedro, Departamento de Neurologia, Niterói RJ, Brazil.

**Keywords:** Polyradiculoneuropathy, Chronic Inflammatory Demyelinating, Cognitive Dysfunction, Quality of Life, Fatigue, Chronic Disease, Polirradiculoneuropatia Desmielinizante Inflamatória Crônica, Disfunção Cognitiva, Qualidade de Vida, Fadiga, Doença Crônica

## Abstract

**Background** Studies were carried out with the objective of evaluating the quality of life (QoL) of patients affected by chronic inflammatory demyelinating polyradiculopathy (CIDP). However, the cognitive issue is still little addressed.

**Objectives** To assess the QoL and cognitive impairment of patients with CIDP and to analyze whether there is a correlation between these parameters.

**Methods** Seven patients with CIDP and seven paired controls were subjected to: mini-mental state examination (MMSE); Montreal cognitive assessment (MoCA); digit symbol replacement/symbol copy test (DSST); fatigue severity scale (FSS); Beck depressive inventory-I (BDI-I), and a short-form of health survey (SF-36).

**Results** The mean age of the participants was 50 years (71.4% male). The MMSE and MoCA had no statistical difference between the groups. Patients showed superior results in the memory domain in the MoCA (5 vs. 2, *p* = 0.013). In the DSST, we observed a tendency for patients to be slower. There was a strong negative correlation between fatigue levels and vitality domain (SF-36). There was no significant correlation between depression levels and QoL, and there was no correlation between depression and the results obtained in the cognitive tests. The patients presented higher levels of depression (15.28 vs. 3.42, *p* < 0.001). A total of 57% had severe fatigue, 28.8% self-reported pain, and 57.1% complained of cramps.

**Conclusion** There was no cognitive impairment in these patients. However, there was a tendency of slower processing speed. To better evaluate the alterations found, a study with a larger number of individuals would be necessary. Chronic inflammatory demyelinating polyradiculopathy affects the QoL of patients in different ways.

## Resumo

**Antecedentes** Estudos foram realizados com o objetivo de avaliar a qualidade de vida (QV) de pacientes acometidos pela polirradiculopatia desmielinizante inflamatória crônica (PDIC). No entanto, a questão cognitiva ainda é pouco abordada.

**Objetivos** Avaliar a QV e o comprometimento cognitivo em pacientes com PDIC bem como se existe correlação entre esses parâmetros.

**Métodos** Sete pacientes com PDIC e sete controles pareados foram submetidos a: miniexame do estado mental (MEEM); avaliação cognitiva de Montreal (MoCA); teste de substituição de símbolo de dígito/cópia de símbolo (DSST); escala de gravidade da fadiga (FSS); *Beck depressive inventory-I* (BDI-I) e um *short-form of health survey* (SF-36).

**Resultados** A média de idade dos pacientes foi de 50 anos (71,4% do sexo masculino). O MMSE e o MoCA não apresentaram diferença estatística entre os grupos. Os pacientes apresentaram resultados superiores no domínio memória do MoCA (5 vs. 2, *p* = 0,013). No DSST, observamos uma tendência de os pacientes serem mais lentos. Houve forte correlação negativa entre os níveis de fadiga e o domínio vitalidade (SF-36). Não houve correlação significativa entre níveis de depressão e QV. Não houve correlação entre depressão e os resultados obtidos nos testes cognitivos. Níveis elevados de depressão foram observados nos pacientes (15,28 vs. 3,42, *p* < 0,001). Um total de 57% apresentou fadiga intensa, 28,8% dor autorreferida, e 57,1% queixam-se de câimbras.

**Conclusão** Não há comprometimento cognitivo nos pacientes estudados. Observamos somente uma tendência de lentificação na velocidade de processamento. Para melhor avaliar as alterações encontradas, será necessário estudo com um número maior de indivíduos. A PDIC afeta de diferentes formas o nível de QV de seus portadores.

## INTRODUCTION

In the last 20 years, much knowledge has been sought regarding the quality of life (QoL) of patients with chronic diseases. In the case of chronic inflammatory demyelinating polyradiculopathy (CIPD), some studies have been carried out with the objective of evaluating how the daily lives of these patients have been affected. 1 2 3 However, little is known about the effect of this peripheral condition of the central nervous system (CNS), resulting in cognitive deficits.

Previously, other groups observed cognitive impairment in patients with CIDP. 4 5 However, the lack of standardized cognitive tests restrains the comparison of studies with this goal.

The present study intends to evaluate patients with CIDP from a referral center, to better understand how the QoL of these patients is affected and whether there are deficits in higher mental processes, such as working memory, executive function, processing speed, and sustained attention, among others.

## METHODS

### Study design and participants

This is an analytical, cross-sectional, observational, case-control study approved by the research ethics committee of the hospital, in agreement with the declaration of Helsinki, regarding research on humans. All CIDP patients were invited to attend the neuromuscular diseases outpatient clinic of Hospital Universitário Antonio Pedro at Universidade Federal Fluminense on a predetermined day. Only those who signed the informed consent form were subjected to the research protocol. In this study, 21 patients were excluded from the total of 28 who attended, due to the associated comorbidities that could generate a confounding factor in the result ( Table 1 ). The diagnosis of CIDP as defined or probable was made according to the European Federation of Neurological Societies and the Peripheral Nerve Society guidelines. 6 The control group (CG) was composed by the companions of patients who did not have neurological diseases or complaints.

**Table 1:** Reasons for exclusion (number of patients)

Type 2 diabetes mellitus (5)	Psychiatric illness (1)
Hereditary neuropathy with liability to pressure palsy (HNPP) (1)	Epilepsy (1)
Guillain-Barré syndrome (1)	Preestablished dementia (1)
Multifocal motor neuropathy (1)	Hypothyroidism (1)
Positive serological test for human T-cell lymphotropic virus (1)	Withdrawal during the research (1)
Complex regional pain syndrome (1)	Age > 65 years (3)
	Charcot-Marie- tooth disease (1)

### Instruments

The patients were interviewed and examined to gather information about the disease. Thereafter, participants completed a neuropsychological test battery consisting of mini-mental state examination (MMSE), digit symbol substitution test associated with the symbol copy test (DSST), and Montreal cognitive assessment (MoCA) to evaluate cognitive abilities. The Beck depressive inventory-I (BDI-I), the 36-item short-form health survey (SF-36), and the fatigue severity scale (FSS) were applied to assess QoL, emotional aspects, and to know if there is some degree of fatigue in CIDP patients. To further corroborate our findings when comparing patient performance with the respective reference means in neuropsychological tests, we performed an additional analysis, in which we compared patient performance directly with the performance of healthy controls (CG).

### Statistical analysis

The collected data were tabulated and later submitted to statistical analysis. The statistical program used was R version 3.6.1. The categorical variables were described through frequency and percentages (sex). For continuous variables (age, test scores), the description was made by means and standard deviation for normal data or median and percentiles (p. 25–75) for data with nonparametric distribution.

The Shapiro-Wilk test was used to determine if the data had a parametric distribution. The Fisher exact test was used to analyze the frequency of failures in each domain of the MMSE and MoCA.

When comparing the average time in DSST operations associated with the symbol copy test, a general linear model (two-way analysis of variance [ANOVA] 2 × 3) with Tukey posthoc was used for equal samples.

To correlate the count on the fatigue score (FSS) with depression measured by the BDI and with age in the group of patients, we used the Pearson correlation test for continuous data of parametric distribution and the Spearman correlation test for nonparametric distribution.

To compare the scores of the MMSE, MoCA, depression (BDI), and quality of life (SF-36) between the groups, the Student *t* -test was performed in case of parametric distribution or Wilcoxon-Mann-Whitney in non-parametric distribution.

Significant *p* -values lower than 0.05 and a 95% confidence interval were considered.

The analysis of the test results was made respecting the scores predetermined by each test. We did not reduce the standard deviation to consider cognitive deficit as performed by some studies, as our CG was a mirror of the patients group, paired by a similar number of individuals, sex, age, and scholarity, in the expectation of not generating possible biases.

## RESULTS

Seven patients and seven controls were included in the study. The mean age of the patients was 43 years old vs. 48 years old in the CG. Most of the patients affected by CIDP were male (71.4%).

When comparing the results of the MMSE between the two groups, there was no statistical difference regarding the total score or its individualized domains separately ( Table 2 ).

**Table 2:** Comparison of mini-mental state examination results

	Patient	Control	
**Domain**	**Median**	**Median**	***p***
Guidance	10	10	0.545
Immediate memory	3	3	0.645
Heads up	5	4	0.126
Evocation memory	2	3	0.223
Language	8	8	1
Visuospatial capability	1	1	0.645
Domain	**Average**	**Average**	***p***
total score	25.28	25.28	0.686

Regarding the MoCA results, there was also no statistical difference between the two groups in the total score or in the respective domains ( Table 3 ). The group of patients showed superior results in the test's memory domain (median 5 vs. 2, *p* = 0.013).

**Table 3:** Comparison of Montreal cognitive assessment results

	Patient	Control	
**Domain**	**Median**	**Median**	***p***
Visuospatial capability	3	3	0.890
Memory	5	2	0.013*
Attention, concentration, and working memory	5	6	0.681
Guidance	6	6	0.171
Domain	**Average**	**Average**	***p***
Executive function	2.14	2.42	0.735
Language	3.71	4.57	0.339
total score	24.14	23.28	0.757

Note: * *p* < 0.05.

The statistical methods used also showed that there were no significant differences between the groups when comparing the MMSE and MoCA of patients and controls (MMSE *p* = 0.757; MoCA *p* = 0.687) (patients *p* = 0.504; controls *p* = 0.129). Therefore, it was not possible to assess which of the instruments, MMSE or MoCA, is more sensitive to identify cognitive decline in this sample.

Similarly, there was no difference between the groups with respect to the results of the digit symbol substitution test (DSST) associated with the symbol copy test (30s *p* = 0.209; 60s *p* = 0.143; 90s *p* = 0.231). We also assessed whether there was a correlation between DSST (90s) and executive function (track test, phonemic verbal fluency, and verbal abstraction). The results were: control group *p* = 0.685, patient group *p* = 0.717. For the comparison of DSST and visuospatial capacity (clock drawing test and cube copy) in the MoCA, the results were: control group *p* = 0.625 and patients' group: *p* = 0.212. Therefore, no significant correlation in any of the correlations.

**Figure 1:**
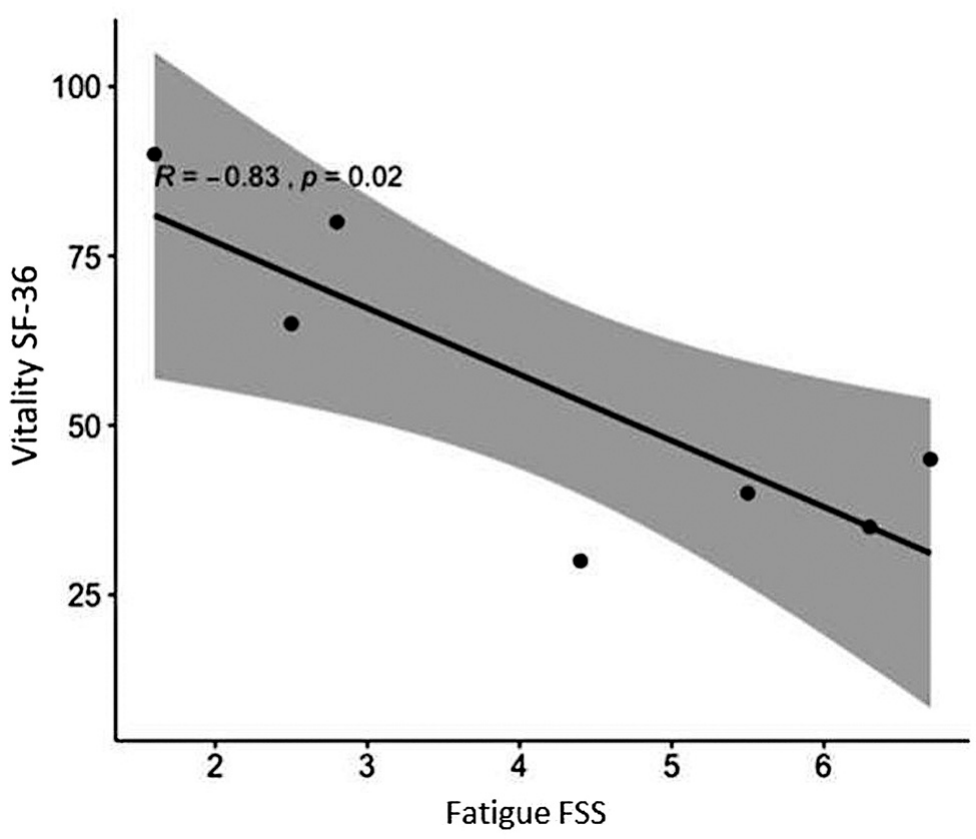
Correlation between fatigue and quality of life – vitality domain.

There was no correlation between the fatigue levels of patients assessed by the FSS and the levels of QoL in most domains of SF-36 ( *p* = 0.610). However, there was a strong negative correlation between fatigue levels and the SF-36 vitality domain (rho = -0.833, *p* = 0.019) ( Figure 1 ). There was also no significant correlation between depression (BDI) or age and patients' quality of life. Just as there was no correlation between fatigue and depression in patients ( *p* = 0.610) ( Table 4 ).

**Table 4:** Correlation between the short-form health survey domains and fatigue, depression, and age

Variable	Functional capacity (p)	Limitation by physical aspects (q)	Pain (p)	General health status (p)	Vitality (p)	Social aspects (p)	Limitations due to emotional aspects (p)	Mental health (p)
Fatigue (FSS)	0.264	0.289	0.213	0.061	0.019* (rho = −0.833)	0.196	0.295	0.090
Depression (BDI)	0.943	0.808	0.321	0.493	0.936	0.393	1	0.507
Age	0.725	0.354	0.953	0.08	0.159	0.708	0.738	0.162

Abbreviations: BDI, Beck depressive inventory; FSS, fatigue severity scale.

Note: * *p* < 0.05

Finally, QoL was assessed comparatively in both groups. The control group showed higher levels of QoL in the domains of mental health, pain, general health, and limitation due to physical aspects. In addition, higher levels of depression were observed in the group of patients when compared to the CG (mean 15.28 vs. 3.42, *p* < 0.001).

## DISCUSSION

The average age and sex of the patients in the present study found similarity with previous studies, in which CIDP patients are on average 50 years old and mostly male. 1 4 7 8 There is still no information to explain the male predominance, 1 4 7 nor any hypothesis. 1 This finding suggests that some characteristics of certain neuropathies are independent of environmental factors and vary according to the population studied. 7

Chronic inflammatory demyelinating polyradiculopathy is a rare disease in which the number of individuals affected is small, including in referral centers for diagnosis. For this reason, we chose not to divide the participants into age and sex subgroups when performing the statistical analyses presented. However, there is sufficient information for discussion about the subject and observation of trends that can be evaluated further in later using larger sample studies. It is relevant to notice that the CG of this study has the same number of individuals belonging to the patients group, in addition to a similar number of men and women and a very close level of education. The average age is also practically the same between the two groups. This fact makes the CG a mirror of the patients in this study and provides a more accurate comparison between them. All of this in the expectation of not generating possible biases, as observed in the study by Yalachkov et al., (2020), in which the CG has a much higher number of individuals than the group studied, in addition to a higher level of education.

In the assessment of the MMSE, there were no results below the reference value for normality according to the predetermined score for the level of education of the population of this study. There are few published studies on the assessment of cognitive deficits in peripheral neuropathies and these with different applied methodologies. 4 5 Thus, the comparison between published papers is impractical.

The results of the MoCA of this population were curious, since the CG obtained a result below the expected for the short-term memory domain. As neither age nor schooling differed significantly between the groups, anxiety for excellent performance on cognitive tests and discomfort with the research environment are likely causes of this result.

This battery of tests, like the MMSE, was not included in the few existing studies on cognitive function in patients with CIDP. 4 5 This fact also makes comparison with their results impossible. Since MoCA is an easy-to-apply battery, it can be performed even during routine outpatient consultations, with high sensitivity for the diagnosis of mild cognitive impairment, it is an indispensable test in any study that aims to assess cognitive function and its particularities.

The information processing speed (IPS) plays an important role in the processes that are requested subsequently as memory, attention, and executive function. Researchers suggest that slow IPS is a characteristic of cognitive impairment. 9

To better evaluate this processing speed, executive functioning, working memory, concentration, and sustained attention, we use the DSST associated with the symbol copy test. Although we did not find statistically significant differences between the study population, analyzing the average result obtained in each test, we observed a tendency for patients to be slower than the CG in carrying out the tests. There is an inclination to the CG be more effective when using the memorization and learning mechanism, causing it to have an average time gain when copying symbols. This did not occur in average values in the group of patients ( Table 5 ). This result was different from those evidenced by the group from Germany. 4 This fact is probably due to a bias in the selection of the CG or by a very small number of individuals in the present study, for whom it was not possible to decrease the standard deviation of the tests, as it is frequently used and is already well established in cognitive assessment research with neurological patients. 4 The execution of the DSST involves the recruitment of frontoparietal areas related to selective attention processes, occipital areas related to visual attention, and cerebellum, demanding both hemispheres for most areas. 10 These outcomes reflect the recruitment of areas related to the downward control of attention, such as the middle and upper frontal gyri and the upper and lower parietal gyri. 11 12

**Table 5:** Results obtained from the digit symbol copy test and symbol copy

**Control**	**Digit symbol**			**Average time spent per symbol**			**Average**
	**30**	**60**	**90**	**30**	**60**	**90**	
**1°**	20	34	51	0.67	0.57	0.57	0.38
**2°**	4	8	15	0.13	0.13	0.17	
**3°**	9	18	23	0.30	0.30	0.26	
**4°**	10	23	43	0.33	0.38	0.48	
**5°**	13	25	33	0.43	0.42	0.37	
**6°**	7	14	19	0.23	0.23	0.21	
**7°**	23	45	58	0.77	0.75	0.64	
**Control**	**Symbol copy**			**Average time spent per symbol**			**Average**
	**30**	**60**	**90**	**30**	**60**	**90**	
**1°**	21	41	62	0.70	0.68	0.69	0.43
**2°**	8	12	16	0.27	0.20	0.18	
**3°**	9	19	27	0.30	0.32	0.30	
**4°**	15	32	46	0.50	0.53	0.51	
**5°**	17	30	42	0.57	0.50	0.47	
**6°**	7	12	19	0.23	0.20	0.21	
**7°**	19	41	56	0.63	0.68	0.62	
**Patient**	**Digit symbol**			**Average time spent per symbol**			**Average**
	**30**	**60**	**90**	**30**	**60**	**90**	
**1°**	10	22	33	0.33	0.37	0.37	**0.33**
**2°**	12	26	36	0.40	0.43	0.40	
**3°**	9	17	25	0.30	0.28	0.28	
**4°**	12	18	31	0.40	0.30	0.34	
**5°**	19	37	44	0.63	0.62	0.49	
**6°**	4	7	12	0.13	0.12	0.13	
**7°**	11	19	30	0.37	0.32	0.33	
**Patient**	**Symbol copy**			**Average time spent per symbol**			**Average**
	**30**	**60**	**90**	**30**	**60**	**90**	
**1°**	14	23	36	0.47	0.38	0.40	**0.33**
**2°**	8	17	26	0.27	0.28	0.29	
**3°**	8	15	21	0.27	0.25	0.23	
**4°**	10	19	26	0.33	0.32	0.29	
**5°**	18	31	51	0.60	0.52	0.57	
**6°**	4	9	17	0.13	0.15	0.19	
**7°**	11	24	33	0.37	0.40	0.37	

Concerning the QoL, it is being considered increasingly important regarding the evaluation of the progression of the disease, treatment, and management of care provided to patients with demyelinating diseases. 13 In the sample analyzed in this study, no correlation was observed between the fatigue levels of the FSS patients and the QoL levels in most SF-36 domains. However, there was a strong negative correlation between fatigue levels and the vitality domain among the patients affected by CIDP in the present study ( Figure 1 ), with 57% reporting severe fatigue.

In patients with neurological diseases, fatigue is different from that reported by other patients, leading to a greater impact on daily life. 14 Our sample demonstrated to believe that vitality and fatigue are present together, belonging to the same context.

There was no significant correlation between the levels of depression measured by the BDI-I and the patients' QoL, just as there was no correlation between depression and the results obtained in the cognitive tests.

**Figure 2:**
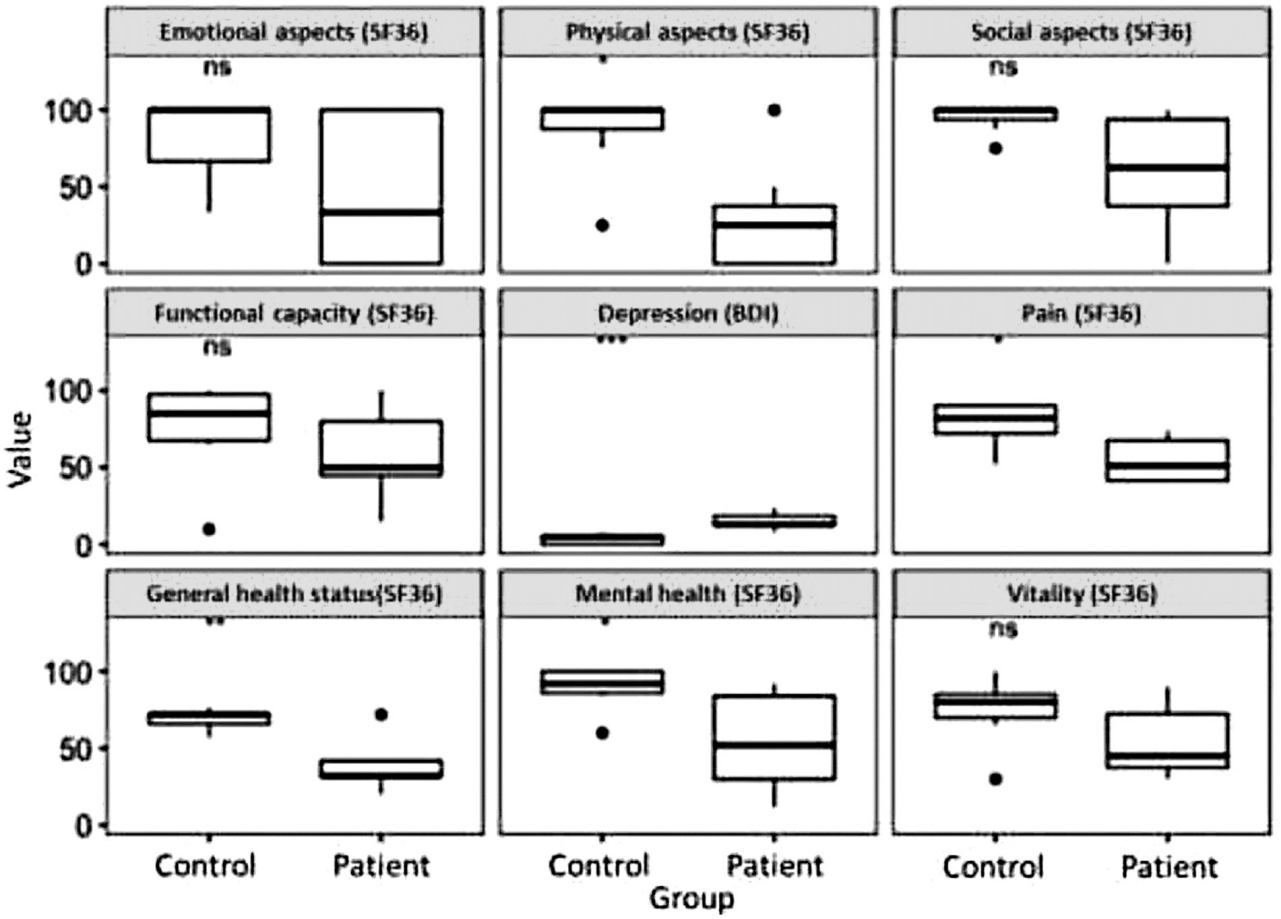
Comparison of the results of the domains of quality of life assessed by the SF-36 and depression by the BDI. Notes: ns: Not significant; * *p* < 0.05; ** *p* > 0.01; *** *p* < 0.001.

**Figure 3:**
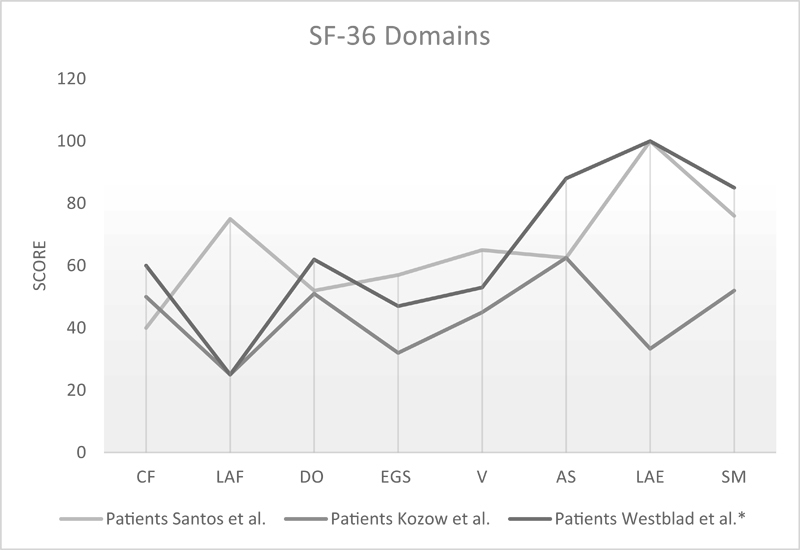
Comparison in the SF-36 domains between studies. Notes: *Approximate data obtained through graphical analysis. **Abbreviations: PC, physical capacity; LFA, limitation by functional aspects; PA, pain; GHS, general health status; V, vitality; SA, social aspects; LEA, limitation by emotional aspects; MH, mental health.

Previous published papers with patients with CIDP or another chronic disease showed that there is a greater impairment of functional capacity and physical health than emotional and social aspects 1 15 ( Figure 2 ). However, in our sample, when we compared the CG with the patients, the CG showed higher QoL in the domains of mental health, pain, general health, and limitation due to physical aspects. The group of patients had higher levels of depression when compared to controls, (average BDI-I score 15.28 vs. 3.42, *p* < 0.001). Although it was not statistically significant, our patients with CIDP had a limitation due to emotional aspects, higher than those evidenced by other groups ( Figure 3 ).

Comparing our findings with other studies on QoL in patients with CIDP, we can see that the affected patients have practically the same level of functional capacity, and that our sample has limitations due to physical aspects similar to that presented by the Swedes. 1 2 It is much lower than what the group from Universidade de São Paulo found in its sample. 1

The SF-36 pain score was very similar in all samples. Nevertheless, only 28.8% of the patients in the present study self-reported pain and 57.1% reported cramps.

Regarding the global health and vitality scale scores, although all samples exhibit similar results, our patients show a worse quality in these domains compared to the other groups. Concerning the mental health domain, the CIDP patients in this study have a worse quality of life in this regard, pointing out that the limitation due to emotional aspects (EAs) is present.

### Study limitations

The small sample size of patients was an important limitation in our study. This compromised the power of the statistical tests. That is, one may not find significant differences due to the type 2 error, in which the alternative hypothesis is rejected, generating a false negative result.

In conclusion, the SF-36 proved to be an excellent instrument for assessing the quality of life of these patients. 1 2 3 As for the cognitive deficit, there were no significant differences between the case group and the control group. Despite this, we verified, through the DSST associated with the symbol copy test, a tendency to decrease the processing speed of patients with CIDP. The application of a broader battery of tests that thoroughly assess the executive function in these individuals may provide more information on this issue. However, due to the small sample size, we could not fill the knowledge gap on cognitive impairment. Chronic inflammatory demyelinating polyradiculopathy is a rare disease and, therefore, it is difficult to acquire many patients and, consequently, have statistical relevance in any study that investigates this pathology. Future multicenter studies, in which a greater number of individuals with CIDP may be present, would be interesting to better assess cognitive impairment in this disease.
